# Reply to: association between stress hyperglycemia on admission and unfavorable neurological outcome in OHCA patients receiving ECPR (https://doi.org/10.1007/s00392-022-02057-4)

**DOI:** 10.1007/s00392-022-02097-w

**Published:** 2022-09-13

**Authors:** Xavier Bemtgen, Tobias Wengenmayer, Dawid L. Staudacher

**Affiliations:** Interdisciplinary Medical Intensive Care (IMIT), Faculty of Medicine, Medical Center-University of Freiburg, Hugstetterstrasse 55, 79106 Freiburg, Germany

## Sirs:

With interest we read the article by Takuya Taira and coauthors on blood glucose levels and outcome in patients undergoing extracorporeal resuscitation (ECPR) [1]. The authors have to be complimented for their efforts in ECPR research. In their retrospective analysis of more than one hundred patients, the authors show a correlation between admission blood glucose levels and neurological outcome. The authors conclude that blood glucose of 200–300 mg/dL (stress hyperglycemia) at admission is associated with favorable neurological outcome after cardiac arrest compared to blood glucose under 200 mg/dL.

But association does not prove causality. A plausible hypothesis on the pathophysiological bases of the observed correlation is important before conclusion can be drawn. The human brain almost exclusively relies on aerobic glycolysis for homeostasis [2] and severe hypoglycemia causes brain damage just like ischemia or hypoxia [3]. Therefore, when discussing a correlation of blood glucose levels with poor neurological outcome, hypoglycemia is an important confounder. The clinical relevance of hypo- as well as hyperglycemia have been elegantly shown for patients after out-of-hospital arrest in a large register study [4]. This U-shaped association of blood glucose at admission on in-hospital mortality is also present in patients with cardiogenic shock [5]. The same correlation between extreme blood glucose levels and prognosis was shown in patients on venoarterial ECMO with and without ECPR [6]. A U-shaped correlation of glucose with adverse outcome is reasonable from a pathophysiological point of view and has been documented in literature.

We, therefore, feel that neglecting hypoglycemia in the present research significantly limits the conclusions that can be drawn from the presented dataset [1]. Clinicians are ill-advised using the suggested cutoff of blood glucose < 200 mg/dL as surrogate for poor prognosis in ECPR. First, we do not know how prognosis would have been without ECPR and second the hypoglycemic bias is unresolved.

## References

[1] Taira T, Inoue A, Nishimura T (2022). Association between stress hyperglycemia on admission and unfavorable neurological outcome in OHCA patients receiving ECPR. Clin Res Cardiol.

[2] Mergenthaler P, Lindauer U, Dienel GA, Meisel A (2013). Sugar for the brain: the role of glucose in physiological and pathological brain function. Trends Neurosci.

[3] Auer RN (2004). Hypoglycemic brain damage. Metab Brain Dis.

[4] Nehme Z, Nair R, Andrew E (2016). Effect of diabetes and pre-hospital blood glucose level on survival and recovery after out-of-hospital cardiac arrest. Crit Care Resusc J Australas Acad Crit Care Med.

[5] Kataja A, Tarvasmäki T, Lassus J (2017). The association of admission blood glucose level with the clinical picture and prognosis in cardiogenic shock—results from the CardShock Study. Int J Cardiol.

[6] Bemtgen X, Rilinger J, Jäckel M (2021). Admission blood glucose level and outcome in patients requiring venoarterial extracorporeal membrane oxygenation. Clin Res Cardiol.

